# Effect of maternal country of birth on breastfeeding practices: results from Portuguese GXXI birth cohort

**DOI:** 10.1186/s13006-018-0157-x

**Published:** 2018-04-10

**Authors:** Musa Abubakar Kana, Carina Rodrigues, Maria João Fonseca, Ana Cristina Santos, Henrique Barros

**Affiliations:** 1 EPIUnit-Instituto de Saúde Pública da Universidade do Porto, Rua das Taipas, 135, 4050-600 Porto, Portugal; 2 Department of Community Medicine, College of Medicine, Kaduna State University, Kaduna, Nigeria; 3 Departamento de Ciências da Saúde Pública e Forenses e Educação Médica, Faculdade de Medicina, Universidade do Porto, Porto, Portugal

**Keywords:** Breastfeeding, Initiation, Duration, Maternal country of birth, Portugal

## Abstract

**Background:**

Maternal country of birth has been associated with perinatal health outcomes but less is known regarding breastfeeding practices in contemporary European settings. This study investigated effect of maternal country of birth on breastfeeding initiation and duration by comparing native Portuguese and migrant mothers.

**Methods:**

We analyzed data of 7065 children of the Generation XXI (GXXI) birth cohort recruited at birth (2005–06) and followed-up 4 years later. Logistic regression was used to assess the effect of maternal country of birth on breastfeeding initiation. Kaplan-Meier estimate was used to compare breastfeeding duration by maternal country of birth and length of residence by migrant mothers in Portugal.

**Results:**

Breastfeeding initiation and the type of breastfeeding practice were similar for native Portuguese and migrant mothers. The migrants had significantly higher median duration in months of any breastfeeding (Odds Ratio [OR] 6.0, 95% Confidence Interval [CI] 5.4,6.6) and exclusive breastfeeding (OR 4.0, 95% CI 3.8,4.2) than native Portuguese mothers (OR 4.0, 95% CI 3.8,4.2 and OR 3.0, 95% CI 2.9,3.0). Migrant mothers who resided in Portugal for either ≤5 years (OR 5.0, 95% CI 3.9,6.1 and OR 4.0, 95% CI 3.8,4.2) or >  5 years (OR 6.0, 95% CI 5.5,6.5 and OR 4.0, 95% CI 3.7,4.3) years had similar duration of any breastfeeding or exclusive breastfeeding, in both cases higher than the native Portuguese mothers. No significant differences were found when world regions were compared.

**Conclusions:**

Maternal country of birth does not influence breastfeeding initiation and type of feeding practice. However, migrant mothers have longer breastfeeding duration of either exclusive or any breastfeeding, which was not changed by length of residence in Portugal.

**Electronic supplementary material:**

The online version of this article (10.1186/s13006-018-0157-x) contains supplementary material, which is available to authorized users.

## Background

Breastfeeding is a universally accepted means of optimal nutrition that promotes infant survival, growth and development [1, 2]. World Health Organization (WHO) recommends that infants be exclusively breastfed for the first 6 months of life with continued breastfeeding up to 2 years of age or beyond with adequate complementary feeds [3]. Breastfeeding is cost effective with life course benefits for mother and child [4]. Fundamentally, it has been established to have a positive effect on intelligence, to be protective against childhood infections and obesity, as well as decreasing risk of some non-communicable diseases in adulthood [4, 5].

Infant feeding practices are determined by sociodemographic factors including maternal country of birth [1]. However, this association is typified by mixed findings [1, 6–9]. There are results indicating that immigrants with high income are more likely to initiate and breastfeed for longer duration than native born mothers [1, 8, 9]. While in other studies lesser or comparable initiation and duration rates were observed irrespective of maternal country of birth [6, 7]. Although the association is not homogenous for all settings, “healthy immigrant effect” could be considered the basis for the disparity of breastfeeding behaviour in migrant and native born mothers [10]. Comparing breastfeeding duration between emigrants and mothers in their home country also suggests a “healthy migrant effect” because of the better indicators in the emigrants [11].

“Healthy immigrant effect” describes immigrants as having better health outcomes compared with native-born and non-migrant populations in countries of origin [10]. The relationship between the maternal country of birth and perinatal outcomes has been well documented, but few regarding breastfeeding [4, 12, 13]. Even fewer studies addressed the effect of duration of residence by migrants in the host country on breastfeeding in European settings [14, 15]. In international terms, the share of immigrants particularly from contemporary conflict zones in total population in Portugal is relatively low. The national statistics shows that the immigration flows into the country, which accelerated in the last three decades is mainly linked with employment opportunities, and females constituted a slightly greater proportion of the immigrant population [16, 17]. Therefore, this study investigated breastfeeding initiation and duration in a birth cohort comparing Portuguese and migrant mothers. Additionally, we examined the effect of time since migration to Portugal on breastfeeding practices. We hypothesized that migrant mothers would have greater proportions of breastfeeding initiation and longer duration.

## Methods

### Study setting and context

Generation XXI (GXXI) is a population-based prospective birth cohort that initially enrolled a total of 8647 newborns between April 2005 and August 2006 from all five public maternity units of the metropolitan area of Porto, Portugal [18]. Information was collected on maternal demographic and obstetric characteristics, as well as parental lifestyle (e.g. diet, smoking, alcohol consumption, physical activity) during the pregnancy and in the early period of baby’s life. Data on a wide range of pregnancy outcomes and information on child health were also obtained. Exposure and outcome data were collected directly from participants by questionnaires, interviews and clinical assessments [18]. Details of the methods including data collection tools and processes are also found in the project Generation XXI website [19].

In this study, we analyzed data from the Generation XXI obtained at birth and 4 years follow-up evaluation [20]. Four years after birth, the total cohort was invited for reevaluation between April 2009 and July 2011.Trained interviewers administered a structured questionnaire concerning maternal health, which included breastfeeding experience. In this study, only children with data on breastfeeding practices obtained at the 4 years evaluation and maternal country of birth (Portuguese, non-Portuguese Europeans, South Americans and Africans) were included. We excluded migrants from any other regions (*n* = 6) and twins (*n* = 296). The final sample included 7065 participants (Additional file 1). Characteristics of participants and non-participants regarding maternal sociodemographic characteristics at birth are presented in Additional file 2.

### Measurements

#### Outcome measures

Type of breastfeeding practices was defined in three categories: exclusive formula when the child never received breast milk; exclusive breastfeeding when the child was only fed with breast milk, and it was considered until formula or another type of foods and beverages were introduced and breastfeeding stopped; and mixed if combined breast milk with formula or other type of foods any time (i.e., concurrently since birth or when the child was exclusively breastfed for a period of time and then formula or another type of foods were introduced to supplement breastfeeding). Any breastfeeding was considered if the child ever received breast milk, regardless if it was exclusive or mixed, categorized as “Yes” (ever breastfed) or “No” (never breastfed). Breastfeeding duration indicated how long the child was exclusively breastfed and also for how long the child was breastfed in total (any type of breastfeeding). Duration of breastfeeding was recorded in weeks and categorized in months. The total duration of breastfeeding was categorized in six groups: never breastfed (0 months), breastfed during < 1 month, breastfed between 1.0 and 2.9 months, between 3.0 and 5.9 months, between 6.0 and 11.9 months, and breastfed for or more than 12 months. These cut-off points were based on WHO recommendations [3]. For exclusive breastfeeding duration we created 5 groups: never exclusively breastfed, breastfed during < 1, exclusively breastfed between 1.0 and 2.9 months, between 3.0 and 5.9 months, and those exclusively breastfed for ≥6.0 months.

#### Exposure measure

A woman was classified as migrant if (a) she was born abroad and both parents were foreign born or (b) she was born abroad and one or both parents were Portuguese born and she moved to Portugal at the age of 18 or later. Otherwise, the participant was considered Portuguese [21]. Time since migration to Portugal was used as a proxy for measuring acculturation and social integration into Portuguese society. We adopted and adapted published classification and assessed the level of acculturation by time since migration to Portugal measured as completed years from arrival to delivery. For analysis we classified duration as ≤5 or >  5 years duration groups as previously done [22].

#### Covariates

Potential confounders considered in analysis included maternal and child specific factors measured at birth: maternal age (< 20, 20–34 and ≥ 35 years), education (basic, secondary or tertiary), employment (employed or unemployed) and household income classified based on euros (€)/month as ≤1000 (low), 1001–1500 (medium) and >  1500 (high), parity (primiparae or multiparae), maternal pre-pregnancy body mass index (BMI < 25, underweight/normal weight or ≥ 25 kg/m^2^, overweight/obese), smoking during pregnancy (smoking or not), number of prenatal visits (≤ 6 prenatal visits or >  6 prenatal visits), gestational age at birth (< 32, 32–36, 37–41 and ≥ 42 weeks), mode of delivery (vaginal or caesarian section). Infants were classified as preterm if delivered at < 37 completed weeks, and low birthweight was defined as babies with birthweight less than 2500 g. While small for gestational age (SGA) babies were classified as those with birthweight below the 10th percentile for the gestational age and sex.

### Statistical analysis

Sociodemographic, obstetric and infant as well as breastfeeding characteristics were described by maternal country of birth (Portugal, other European countries, South American and African). Proportions were compared using the Pearson’s chi-square or Fisher’s exact test for categorical variables. For immigrant mothers, we also described their length of residence in Portugal (≤ 5 vs. > 5 years) and age on arrival (< 18 or ≥ 18 years). We compared breastfeeding practices for exclusive and any breastfeeding. Median duration of exclusive or any breastfeeding were compared using Kruskal-Wallis test. Analyses of any breastfeeding and exclusive breastfeeding duration (continuous variable) excluded never breastfed and outlier observations (exclusive breastfeeding > 36 weeks and for any breastfeeding > 180 weeks).

Logistic regression analysis was used to estimate the effect of maternal country of birth (Portuguese vs. migrants) on breastfeeding initiation (any or exclusive categorized as “Yes” or “No”) expressed as OR (95% CI). Multiple regression analysis was performed to adjust for age, education, household income, parity, pre-pregnancy BMI, number of prenatal visits, smoking during pregnancy, mode of delivery, gestational age, birthweight and Neonatal Intensive Care Unit (NICU) admission. In all the models, native Portuguese was considered as reference category for comparison with other Europeans, South Americans and Africans. Kaplan-Meier estimate was used to compare breastfeeding duration by maternal country of birth and length of residence by migrant mothers in Portugal. The log-rank test was used to compare median breastfeeding duration (95% CI) of Portuguese and migrants mothers. A *p -* value < 0.05 was considered statistically significant. All statistical analyses were performed using Statistical Package for Social Sciences (SPSS), version 23.0 (IBM Corp., New York, USA).

## Results

Participants consisted of native Portuguese (6831, 96.7%), other Europeans (61, 0.9%), South Americans (106, 1.5%) and Africans (67, 0.9%) (Table 1). There is variation in some observed characteristics of foreign and native born mothers. The majority (80.2%) of South Americans had resided for ≤5 years in Portugal as compared to 39.3 and 29.9% of other Europeans and Africans. The proportion of migrants that arrived in Portugal after ≥18 years of age was: other Europeans (93.4%), South Americans (97.2%) and Africans (70.1%). Mothers aged ≥35 years were around one third among Africans (31.3%) but lower percentages for South Americans (14.2%), other Europeans (8.2%) and native Portuguese (17.1%). Only 21.4% of Portuguese mothers and from 17.9 to 39.3% of migrants were tertiary educated. The proportion of households earning 1000 euros or less per month were 38.4% for native Portuguese, 24.5% for other Europeans, 52.3% for South Americans and 42.1% for Africans. The proportion of primiparous mothers was 57.5% (native Portuguese), 73.8% (other Europeans), 62.5% (South Americans) and 48.8% (Africans). More than 80% of mothers attended > 6 prenatal visits irrespective of their country of birth. Caesarean section rates were 35.9% (native Portuguese), 33.3% (other Europeans), 51.9% (South Americans) and 37.9% (Africans). Low birthweight prevalence was 7.1% (native Portuguese), 3.3% (other Europeans), 3.8% (South Americans) and 4.5% (Africans). Preterm birth and NICU admission proportions were respectively 7.2 and 8.7% for native Portuguese, 6.6 and 19.3% for other Europeans, 4.7 and 4.0% for South Americans, 9.0 and 6.1% for Africans.

**Table 1:** Sociodemographic, obstetric, infant and breastfeeding characteristics by maternal country of birth

Characteristic, *n* (%)	Portuguese	European	South American	African	*p* - value
Median length of maternal residence in Portugal (Years)		7.0	3.0	10.5	< 0.05
≤ 5 years		24 (39.3)	85 (80.2)	20 (29.9)	< 0.05
> 5 years		37 (60.7)	21 (19.8)	47 (70.1)	
Age at arrival in Portugal					
< 18 years		4 (6.6)	3 (2.8)	20 (29.9)	< 0.05
≥ 18 years		57 (93.4)	103 (97.2)	47 (70.1)	
Maternal age at birth (years)					
< 20	330 (4.8)	2 (3.3)	1 (1.0)	0 (0.0)	< 0.05
20–34	5330 (78.0)	54 (88.5)	90 (84.9)	46 (68.7)	
≥ 35	1171 (17.1)	5 (8.2)	15 (14.2)	21 (31.3)	
Marital status					
Married/committed	6418 (94.0)	59 (98.3)	100 (94.3)	60 (89.6)	0.428
Single	405 (6.0)	1 (1.7)	6 (5.7)	7 (10.4)	
Maternal education					
Basic	3287 (48.1)	6 (9.8)	16 (15.1)	18 (26.9)	< 0.05
Secondary	2081 (30.5)	31 (50.8)	71 (67.0)	29 (43.3)	
Tertiary	1463 (21.4)	24 (39.3)	19 (17.9)	20 (29.9)	
Maternal occupation					
Employed	5354 (79.1)	50 (82.0)	74 (69.8)	47 (71.2)	0.076
Unemployed	1413 (20.9)	11 (18.0)	30 (30.2)	19 (28.8)	
Household income (euros/month)					
≤ 1000	2289 (38.4)	13 (24.5)	46 (52.3)	24 (42.1)	< 0.05
1001–1500	1776 (29.9)	18 (34.0)	17 (19.3)	10 (17.5)	
> 1500	1894 (31.8)	22 (41.5)	25 (28.4)	23 (40.4)	
Primiparae	3866 (57.5)	45 (73.8)	65 (62.5)	30 (48.8)	< 0.05
Overweight/Obese	2042 (29.9)	10 (16.4)	27 (25.5)	14 (20.9)	
> 6 prenatal visits	5848 (89.8)	51 (86.4)	84 (84.0)	53 (84.1)	0.096
Maternal smoking during pregnancy	1512 (22.1)	17 (27.9)	16 (15.1)	8 (11.9)	< 0.05
Caesarean section delivery	2445 (35.9)	20 (33.3)	54 (51.9)	25 (37.9)	< 0.05
Infant’s sex (male)	3346 (49.0)	26 (42.6)	55 (51.9)	34 (50.7)	0.702
Preterm infant	493 (7.2)	4 (6.6)	5 (4.7)	6 (9.0)	0.721
Low birthweight infant	484 (7.1)	2 (3.3)	4 (3.8)	3 (4.5)	0.242
Small for gestational age infant	1006 (14.7)	6 (9.8)	11 (10.4)	4 (6.0)	0.081
NICU Admission	442 (8.7)	9 (19.3)	3 (4.0)	3 (6.1)	< 0.05
Type of breastfeeding practices					
Exclusive formula	446 (7.4)	2 (3.9)	6 (6.4)	3 (5.1)	0.841
Exclusive breastfeeding	1353 (22.4)	12 (23.5)	21 (22.3)	10 (16.9)	
Mixed feeding	4232 (70.2)	37 (72.5)	67 (71.3)	46 (78.0)	
Duration of exclusive breastfeeding in months					
< 1 month	965 (17.6)	6 (12.5)	8 (9.6)	4 (7.5)	< 0.05
1.0–2.9 months	1354 (24.7)	8 (16.7)	16 (19.0)	11 (20.4)	< 0.05
3.0–5.9 months	2258 (41.2)	25 (52.1)	37 (44.0)	21 (38.9)	
≥ 6.0 months	902 (16.6)	9 (18.8)	23 (27.4)	18 (33.3)	
Total duration of any breastfeeding in months					
< 1 month	497 (5.8)	3 (7.9)	4 (5.9)	2 (4.2)	< 0.05
1.0–2.9 months	740 (16.6)	2 (5.3)	6 (8.8)	3 (6.3)	
3.0–5.9 months	867 (19.4)	8 (21.1)	11 (16.4)	7 (14.6)	
6.0–11.9 months	1309 (29.3)	13 (34.2)	29 (42.6)	11 (22.9)	
≥ 12 months	1157 (25.9)	12 (31.6)	18 (26.5)	25 (52.1)	

*P*-values are for comparisons of all categories; Some variables contain missing values or excluded outliers (calculation of median duration of exclusive or any breastfeeding)

### Prevalence of breastfeeding initiation and duration

Comparing mothers according to maternal country of birth we observed similarity in their breastfeeding options, considering exclusive formula, exclusive breastfeeding and mixed feeding (Table 1): native Portuguese (7.4, 22.4 and 70.2%), other Europeans (3.9, 23.5 and 72.5%), South Americans (6.4, 22.3 and 71.3%) and Africans (5.1, 16.9 and 78.0%) respectively.

The median duration of any breastfeeding and exclusive breastfeeding was significantly higher for migrant mothers compared to native Portuguese (24.0 and 12.0 weeks), other Europeans (34.0 and 16.0 weeks), South Americans (32.0 and 16.0 weeks) and Africans (50.6 and 16.0 weeks). The proportion of migrant mothers that exclusively breastfed for ≥6.0 months were significantly higher: 16.6% of native Portuguese, 18.8% of other Europeans, 27.4% of South Americans and 33.3% of Africans. Prevalence of any breastfeeding for ≥12 months was 25.9% for native Portuguese, 31.6% for other Europeans, 26.5% for South Americans and 52.1% for Africans.

### Effect of maternal country of birth on breastfeeding initiation and duration

Maternal country of birth was not found to be associated with initiation of any breastfeeding and exclusive breastfeeding (Table 2). Using native Portuguese as the reference category, the adjusted odds ratio (AOR) and 95% Confidence Interval) for any breastfeeding and exclusive breastfeeding were as follows for the various categories of migrants: other Europeans (AOR 0.5, 95% CI 1.9,2.8 and AOR 1.1, 95% CI 0.5,2.2), South Americans (AOR 1.9 95% CI 0.5,7.9) and Africans (AOR 0.9, 95% CI 0.3,2.8 and AOR 1.1, 95% CI 0.5,2.2).

**Table 2:** Effect of maternal country of birth on breastfeeding initiation

	Crude model	Adjusted model (95% CI)*
Any breastfeeding		
Portuguese	1	1
Non-Portuguese European	0.6 (0.2,1.8)	0.6 (0.2,2.9)
South American	2.2 (0.5,9.0)	1.9 (0.5,7.6)
African	0.9 (0.3,3.2)	0.9 (0.3,2.9)
Exclusive breastfeeding		
Portuguese	1	1
Non-Portuguese European	1.2 (0.6,2.4)	1.1 (0.5,2.3)
South American	1.0 (0.6,1.8)	0.9 (0.5,1.7)
African	1.2 (0.6,2.4)	1.1 (0.6,2.2)

Any breastfeeding is mixed feeding or exclusive breastfeeding

^*^Adjusted for age, education, household income, parity, BMI, number of prenatal visits, smoking in pregnancy, mode of delivery, gestational age, birthweight, and NICU admission

**Fig. 1:**
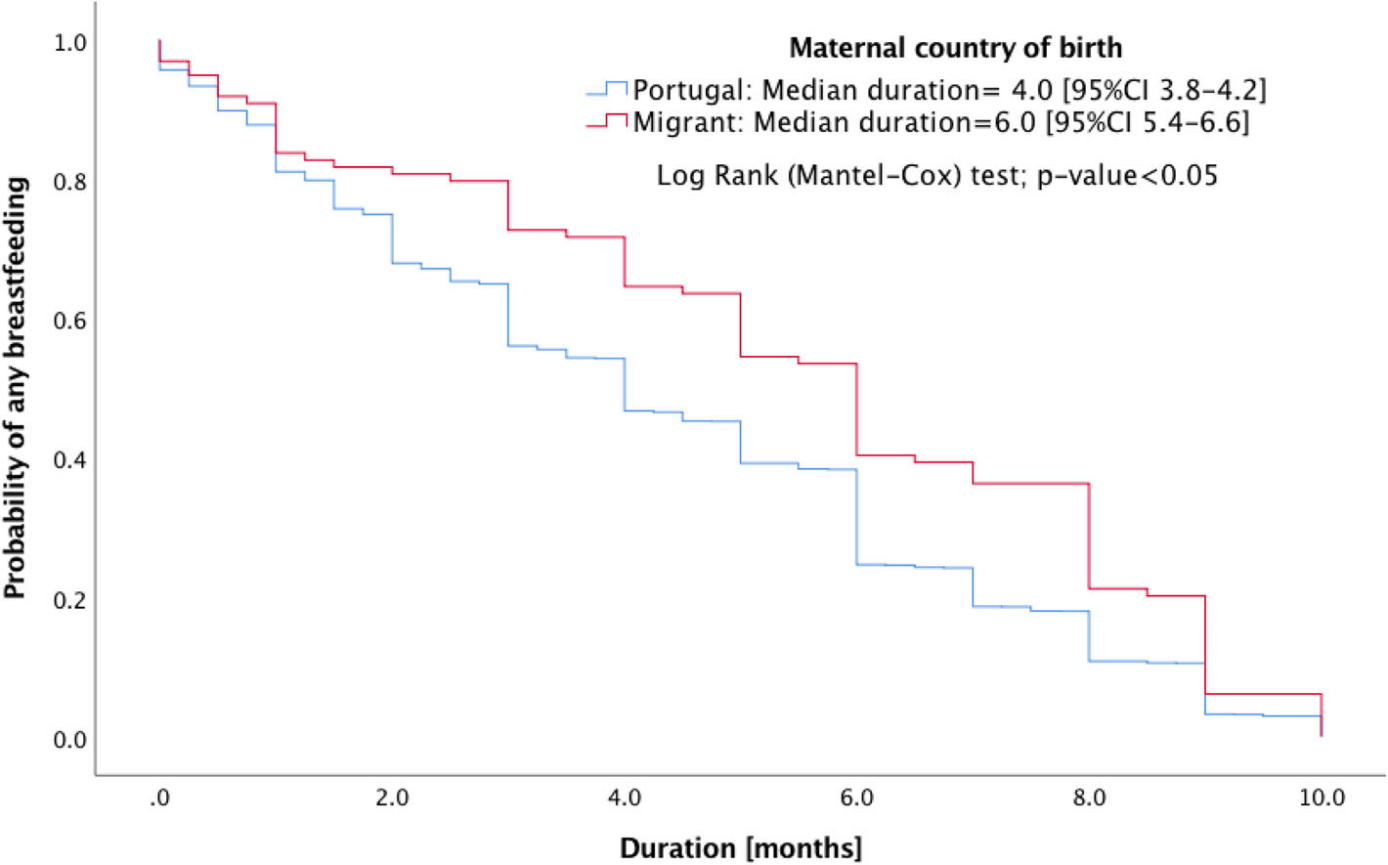
Kaplan-Meier survival curves for duration of any breastfeeding according to maternal country of birth

**Fig. 2:**
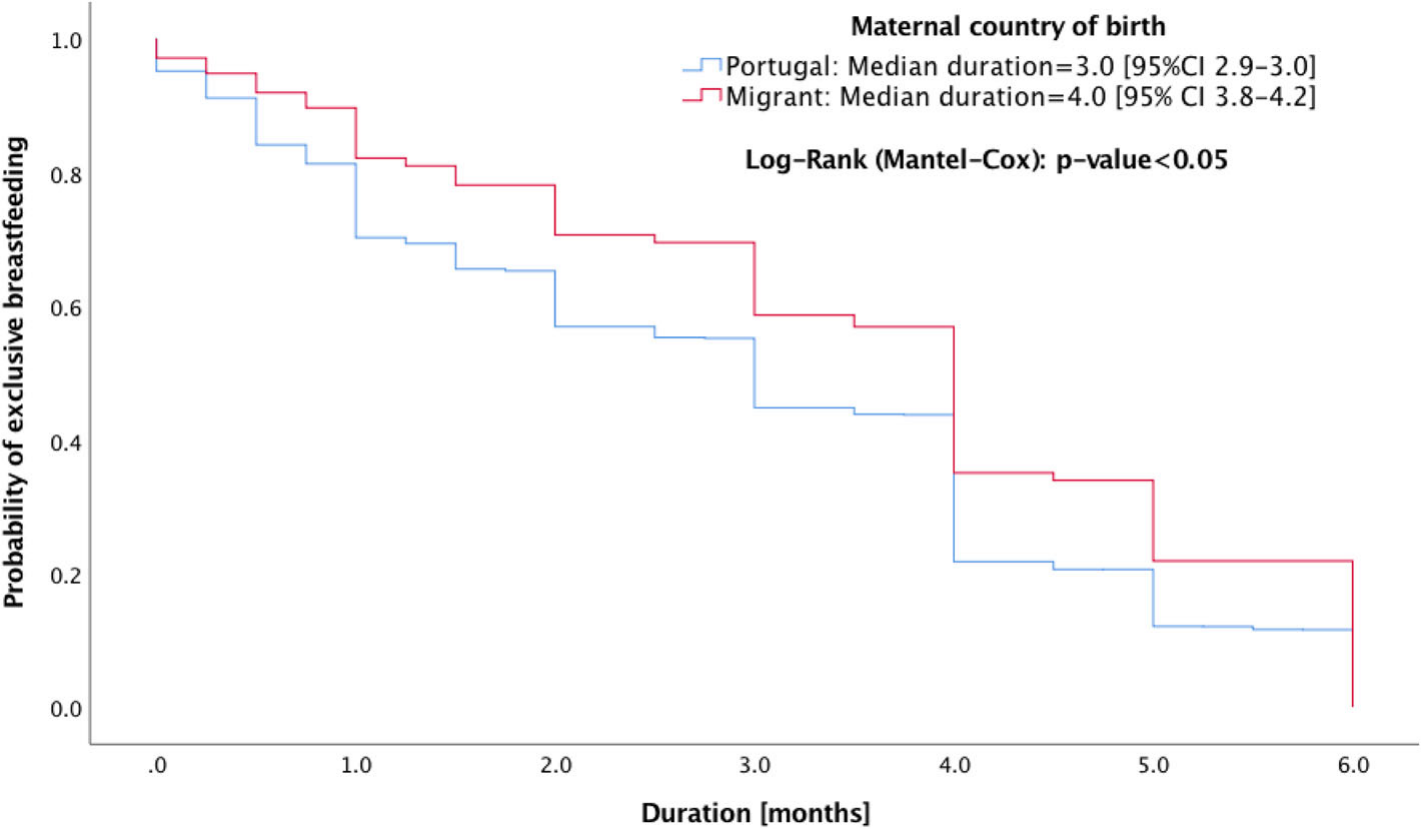
Kaplan-Meier survival curves for duration of exclusive breastfeeding according to maternal immigration country of birth

**Fig. 3:**
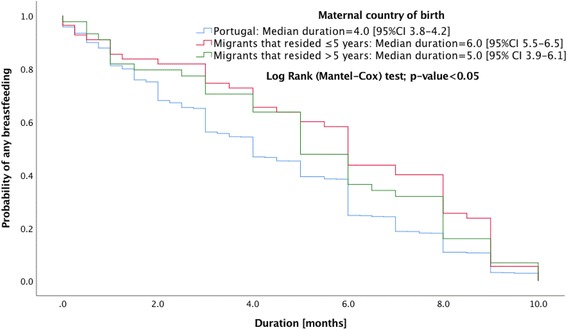
Kaplan-Meier survival curves for duration of any breastfeeding by length of residence in Portugal by migrant mothers (≤5 or > 5 years) compared to native Portuguese mothers

**Fig. 4:**
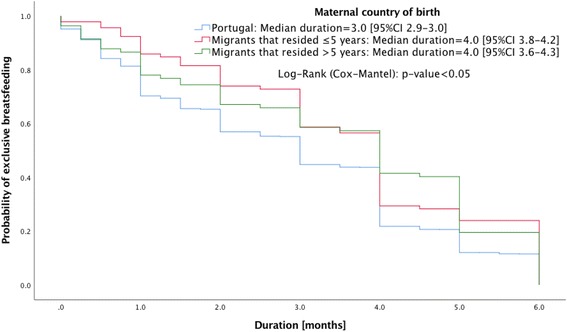
Kaplan-Meier survival curves for duration of exclusive breastfeeding by length of residence in Portugal by migrant mothers (≤5 or > 5 years) compared to native Portuguese mothers

Migrant mothers have significantly higher duration in months of any breastfeeding and exclusive breastfeeding than the native Portuguese (Figs. 1 and 2). Portuguese (OR 4.0, 95% CI 3.8,4.2 and OR 3.0, 95% CI 2.9,3.0), migrants (OR 6.0, 95% CI 5.4,6.6 and OR 4.0, 95% CI 3.8,4.2). Migrant mothers who resided in Portugal for either ≤5 or > 5 years were also observed to have greater duration of any breastfeeding or exclusive breastfeeding compared with native Portuguese mothers (Figs. 3 and 4): migrants who resided ≤5 years (OR 5.0, 95% CI 3.9,6.1 and OR 4.0, 95% CI 3.8,4.2), and migrants who resided > 5 years (OR 6.0, 95% CI 5.5,6.5 and OR 4.0, 95% CI 3.7,4.3).

## Discussion

This study assessed the effect of maternal country of birth on breastfeeding practices among mothers of a birth cohort recruited in Portugal. We observed that the native Portuguese and migrant mothers were similar in their marital status, occupation, number of prenatal visits and birth outcomes. However, there was variation in the distribution of maternal age, education, household income, parity and caesarean section. In our sampled population, the migrants had a higher proportion of tertiary educated mothers. A third of the African born mothers were ≥ 35 years, while the other migrant groups had a lower proportion of this age category compared with the Portuguese. Nevertheless, the proportion of those that initiated any and exclusive breastfeeding were similar for foreign and native born mothers. However, migrants have longer duration of any and exclusive breastfeeding. Furthermore, the length of residence in Portugal by migrant mothers does not negatively impact on the breastfeeding duration.

Breastfeeding could be dependent on the social context of study country or setting. In western developed societies there are evolving social and lifestyle factors that modify breastfeeding practices over time [23]. Substantial proportions of mothers participating in this study were employed and discontinue exclusive breastfeeding before the recommended 6 months duration. This is because maternal employment is known to significantly affect breastfeeding duration [24]. We observed a decline of exclusive breastfeeding at 4 months for both maternal categories, which coincides with the expiration of 4 months of full paid maternity leave according to Portuguese law. [25]

Our findings showed that migrant mothers irrespective of their length of residence in Portugal breastfed (any breastfeeding or exclusive breastfeeding) for longer periods than their native peers, which is contrary to the observation of some studies [15, 26, 27]. Specifically, the African-born women averagely had lengthier period of residence in Portugal but breastfeed for longer duration compared with other migrant mothers. This negates the notion that acculturating to host country could negatively influence migrant mother’s breastfeeding practices, especially those coming from settings with higher breastfeeding rates than Western nations [26]. Our observation is not unusual because acculturation has been reported as not having an influence on breastfeeding for particular migrant groups [7, 28]. Basically, certain traditional beliefs facilitate positive breastfeeding practices that persist for longer period especially in migrants from cultural backgrounds, which western breastfeeding practices was unknown or perceived as unacceptable [29]. On the contrary, it has been shown that post migration, immigrant breastfeeding rates tends to converge towards native born rates of breastfeeding as length of residence in host country increases [15].

An important public health implication of this study is that the duration of any breastfeeding and exclusive breastfeeding were below the WHO recommendations in native Portuguese and migrant mothers. Thus, we recommend intensive breastfeeding promotion for all maternal categories especially for the employed to whom adequate conditions to continue breastfeeding should be provided. Breastfeeding education during prenatal and postnatal care should be emphasized because of its effectiveness at enhancing breastfeeding initiation and duration [30]. In Portugal, there is universal healthcare for women irrespective of immigration status. This is relevant for immigrants because migration isolates women but health professionals could serve as a major source of information on breastfeeding [31]. More so for recently arrived migrant mothers arriving at a time characterized by multiple challenges to breastfeeding practices in contemporary European context. Finally, future research can improve the understanding of the exact role of maternal country of birth in breastfeeding. It is fundamental to explore acculturation pathway to explain healthy migrant effects by comparing breastfeeding practices in source countries with that of first and second generation migrant as well as native-born mothers.

### Strengths and limitations

A major strength of the study is the comprehensive birth cohort data derived for the same mothers at birth and at 4 years evaluation. Even though, recall bias is a potential limitation, it has been reported that maternal recall of infant feeding practices is reliable even after 10 years [32]. The total sample size of this study is large but the proportion of migrants is comparably smaller, which we recognized as a limitation. This poses a challenge for external validity and generalization of findings. However, this was our finding in the study setting, regarding the proportion of migrants, as it was comparable to migrant categories in the contemporary national Portuguese birth registry at inception of the birth cohort. Contextually, the GXXI birth cohort represents 54.6% of all recorded births in greater Porto metropolitan area during recruitment period.

Even though we are unable to study the breastfeeding experience of migrants from some regions like Asia and North America, which were not represented in the study population, we explored migrant diversity by categorizing migrant mothers into three groups based on geographical regions that are predominantly represented in the north of Portugal (other Europeans, South Americans and Africans). We also categorized migrant mothers according to length of residence in Portugal, which is identified as proxy of acculturation in characterizing healthy migrant effect [15].

The data we used for this study has been previously validated. Information about feeding practices was collected from a sub sample of the study population at 6, 15 and 24 months, which was found to be reliable [33]. The analytical approach we employed was also robust in addressing the research question. We evaluated the effect of maternal country of birth on breastfeeding duration by Kaplan Meier survival analysis. This method is effective in estimating time to event (e.g. cessation of breastfeeding) and measuring the fraction of subjects breastfed for a certain amount of time after the initiation of breastfeeding [34]. Kaplan-Meier analysis allows estimation of survival over time, even when participants drop out or are studied for different lengths of time.

## Conclusions

The study has shown that maternal country of birth influences breastfeeding duration but not its initation among mothers of Generation XXI birth cohort. Migrant mothers have longer duration of exclusive or any breastfeeding, which was not changed by length of residence in Portugal. The duration of breastfeeding is below recommended duration in both native and foreign-born mothers. We recommend intensive promotion of breastfeeding for all categories of mothers especially the employed to whom adequate condition to continue breastfeeding should be provided.

## Additional files

Additional file 1 Participant selection flowchart. (DOC 79 kb)

Additional file 2 Comparison between participants and non-participants regarding children and maternal characteristics at birth. (DOC 46 kb)
