# Supplementary figures and images for: Effect of maternal country of birth on breastfeeding practices: results from Portuguese GXXI birth cohort
Source: Int Breastfeed J. 2018 Apr 10;13:15. doi: 10.1186/s13006-018-0157-x (PMC5891910; doi:10.1186/s13006-018-0157-x)

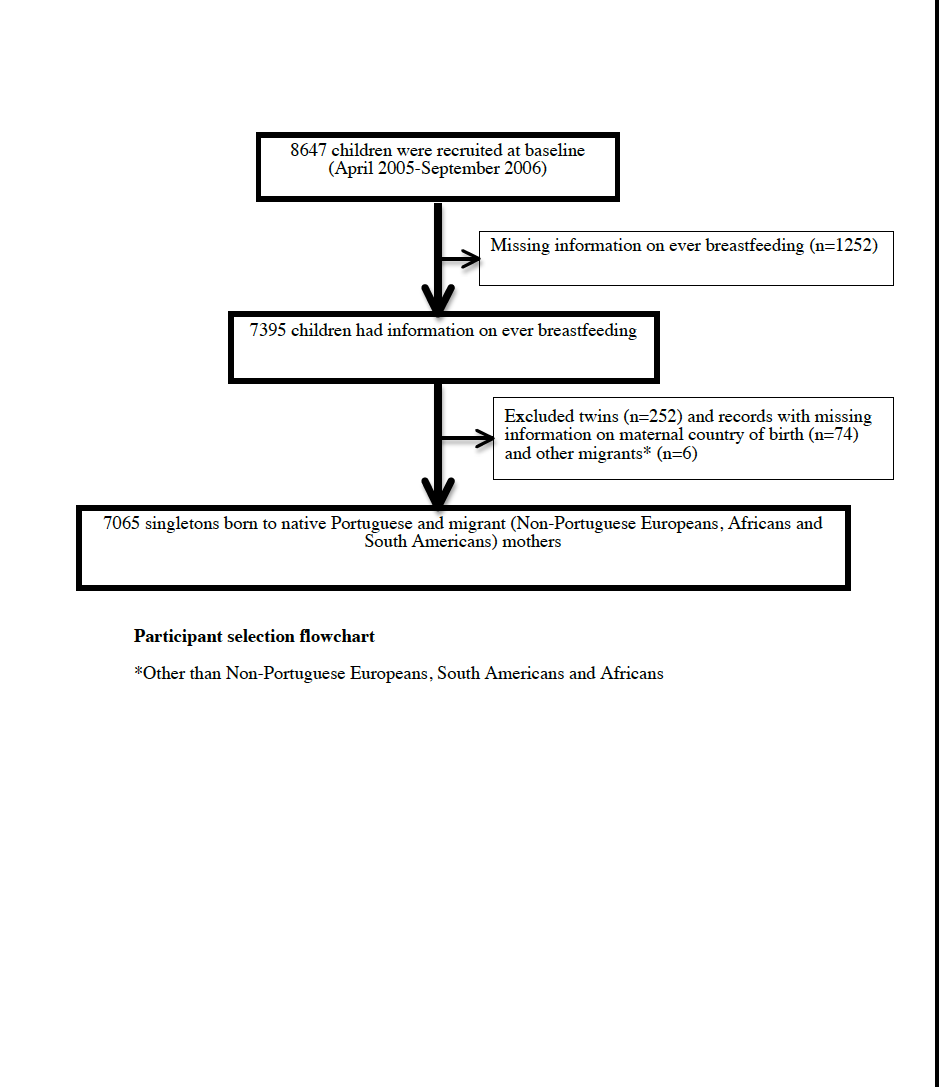

Supplement: Supplementary file 1 — Participant selection flowchart. (DOC 79 kb) [file 13006_2018_157_MOESM1_ESM.doc]
