# Supplementary material for: Effect of maternal country of birth on breastfeeding practices: results from Portuguese GXXI birth cohort
Source: Int Breastfeed J. 2018 Apr 10;13:15. doi: 10.1186/s13006-018-0157-x (PMC5891910; doi:10.1186/s13006-018-0157-x)
Supplement: Supplementary file 2 — Comparison between participants and non-participants regarding children and maternal characteristics at birth. (DOC 46 kb) [file 13006_2018_157_MOESM2_ESM.doc]

**Comparison between participants and non-participants regarding children and maternal characteristics at birth**

| **Characteristic, n (%)** | **Participants, 7065 (85.6)** | **Non-Participants, 1192 (14.4)** | **P-value** |
| --- | --- | --- | --- |
| **Maternal country of birth** |  |  |  |
| Portuguese | 6831 (96.7) | 1077 (90.4) | <0.0005 |
| Non-Portuguese Europeans | 61 (0.9) | 23 (1.9) |  |
| South American | 106 (1.5) | 71 (6.0) |  |
| African | 67 (0.9) | 21 (1.8) |  |
| **Maternal duration of residence in Portugal** |  |  |  |
| ≤5 years | 129 (55.1) | 89 (77.4) | <0.0005 |
| >5 years | 105 (44.9) | 26 (22.6) |  |
| **Maternal age at birth (years)** |  |  |  |
| <20 | 333 (4.7) | 132 (11.1) | <0.0005 |
| 20-34 | 5520 (78.1) | 919 (77.1) |  |
| ≥35 | 1212 (17.2) | 141 (11.8) |  |
| **Maternal education** |  |  |  |
| Basic | 3327 (47.1) | 750 (62.9) | <0.0005 |
| Secondary | 2212 (31.3) | 305 (25.6) |  |
| Tertiary | 1526 (21.6) | 137 (11.5) |  |
| **Family income (euros/month)** |  |  |  |
| ≤1000 | 2372 (38.5) | 527 (53.8) | <0.0005 |
| 1001-1500 | 1821 (29.6) | 226 (23.1) |  |
| >1500 | 1964 (31.9) | 226 (23.1) |  |
| **Type of delivery** |  |  |  |
| Vaginal | 4499 (63.9) | 819 (68.9) | 0.001 |
| Caesarean section | 2544 (36.1) | 370 (31.1) |  |
| **Infant’s sex** |  |  |  |
| Male | 3604 (51.0) | 615 (51.6) | 0.710 |
| Female | 3461 (49.0) | 577 (48.4) |  |
| **Preterm birth** | 508 (7.2) | 110 (9.2) | 0.013 |
| **Low birthweight** | 493 (7.0) | 99 (8.3) | 0.100 |
